# Increase in Lassa Fever Cases in Nigeria, January–March 2018

**DOI:** 10.3201/eid2505.181247

**Published:** 2019-05

**Authors:** Elsie A. Ilori, Christina Frank, Chioma C. Dan-Nwafor, Oladipupo Ipadeola, Amrei Krings, Winifred Ukponu, Oboma E. Womi-Eteng, Ayodele Adeyemo, Samuel K. Mutbam, Emmanuel O. Musa, Clement L.P. Lasuba, Wondimagegnehu Alemu, Sylvanus Okogbenin, Ephraim Ogbaini, Uche Unigwe, Emeka Ogah, Robinson Onoh, Chukwuyem Abejegah, Olufemi Ayodeji, Chikwe Ihekweazu

**Affiliations:** Nigeria Centre for Disease Control, Abuja, Nigeria (E.A. Ilori, C.C. Dan-Nwafor, O. Ipadeola, W. Ukponu, O.E. Womi-Eteng, C. Ihekweazu);; Robert Koch Institute, Berlin, Germany (C. Frank, A. Krings);; African Field Epidemiology Network, Abuja (C.C. Dan-Nwafor);; US Centers for Disease Control and Prevention, Abuja (O. Ipadeola);; University of Maryland, Abuja (W. Ukponu);; eHealth Africa, Abuja (A. Adeyemo);; World Health Organization Abuja Country Office, Abuja (S.K. Mutbam, E.O. Musa, C.L.P. Lasuba, W. Alemu);; Irrua Specialist Teaching Hospital, Edo, Nigeria (S. Okogbenin, E. Ogbaini); F; ederal Teaching Hospital, Abakiliki, Nigeria (U. Unigwe, E. Ogah, R. Onoh);; Federal Medical Centre, Owo, Nigeria (C. Abejegah, O. Ayodeji)

**Keywords:** Lassa fever, outbreak, Lassa virus, viruses, Nigeria, Edo, Ondo, Ebonyi, surveillance, epidemiology, zoonoses, hemorrhagic fever, surveillance capacity

## Abstract

We reviewed data pertaining to the massive wave of Lassa fever cases that occurred in Nigeria in 2018. No new virus strains were detected, but in 2018, the outbreak response was intensified, additional diagnostic support was available, and surveillance sensitivity increased. These factors probably contributed to the high case count.

A massive wave of laboratory-confirmed cases of Lassa fever occurred in Nigeria in 2018. Whether this high case count was caused by a new virus variant, increased seasonal incidence, improved case recognition, availability of laboratory diagnostics and therapy, or a combination of these factors is unknown. We set out to determine the factors that contributed to this outbreak using data available through the Nigerian Disease Surveillance System.

Lassa fever is endemic in Nigeria and peaks during the first 12 weeks of the year (January–March; Figure) (https://ncdc.gov.ng/themes/common/files/sitreps/b7cd08c8047e52ceabb09e5318a3b0a7.pdf). A total of 107 laboratory-confirmed cases were reported during the first 12 weeks of 2017 and 394 during the same period in 2018. Among confirmed and probable cases, 50 deaths were reported during the peak season in 2017 and 104 in 2018. In the neighboring states of Edo and Ondo, 45.0% of confirmed cases occurred in 2017 and 66.0% in 2018. These states have access to the long-established and largest Lassa fever treatment center that has Lassa virus diagnostic capabilities in Nigeria. Although Edo and Ondo comprise only 4.6% of Nigeria’s population, the population in these states accounted for 74.6% of the increase in confirmed cases during the 2017 and 2018 peak seasons. Another area with a strong increase in case numbers in 2018 was the nonadjacent Ebonyi State, where another laboratory was outfitted to conduct Lassa virus diagnostics by early 2018.

**Figure:**
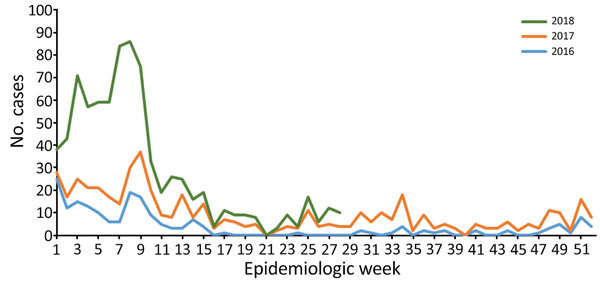
Weekly trends of confirmed Lassa fever cases, Nigeria, 2016–2018.

Evidence from sequencing studies yielded no indication that viruses circulating in 2018 were different from those found in previous years (*1*). Phylogenetic data suggest multiple zoonotic infections instead of extensive person-to-person transmission.

The increased case numbers in Edo, Ondo, and Ebonyi could not be accounted for by gross population changes; these states are distant from the refugee emergency in northeastern Nigeria (http://reporting.unhcr.org/node/21275). Studies on climatic and other environmental circumstances (e.g., new agricultural practices or larger harvests) that could have potentially contributed to changes in the way and frequency humans come into contact with the Lassa virus reservoir, *Mastomys natalensis* mice, have not been established. Also, data on rodent abundance are not available.

However, in 2018, many changes occurred in Nigeria regarding Lassa virus surveillance and treatment. Instead of only 1 Lassa virus laboratory for Edo and Ondo and 1 in Lagos, 2 additional laboratories (in Ebonyi and Abuja) started providing diagnostic services in early 2018. Sample transport logistics had improved, and patients’ samples were transported and tested without charge. Considering that laboratory confirmation is a prerequisite for counting cases, laboratory capacity building directly affects surveillance case numbers. By early 2018, Lassa fever surveillance had been strengthened by the formulation of standard operating procedures, and rapid response teams provided capacity building and support to affected states 4 weeks earlier than they did in 2017. Clinical cases of Lassa fever need to be treated in isolation facilities, and by 2018, additional treatment centers had been assessed and were being supported by the federal government. At this time, ribavirin was also available to patients without charge, and the media actively advertised this message, encouraging patients to seek medical treatment. In addition, Lassa fever received strong media attention in early January 2018 because of an incident in a healthcare facility where 3 of 4 infected healthcare workers died.

Surveillance data are incomplete for 2018, but higher surveillance sensitivity is visible in the available data from the peak 2017 and 2018 seasons. The percentage of suspected Lassa fever cases testing positive decreased from 31.0% in 2017 to 21.6% in 2018 (30.2% decrease). Nosebleed, an indicator of disease severity in nonfatal confirmed cases, was noted on 10.8% of case report forms for surviving patients in 2017, but this frequency declined to 2.6% in 2018 (75.9% decrease), and the case-fatality ratio among confirmed and probable cases declined from 43.9% in 2017 to 25.8% in 2018 (41.2% decrease). Disease severity and case-fatality ratios are also influenced by the timeliness of patients seeking treatment and treatment availability. The decrease in the 3 aforementioned factors (percentage of Lassa virus–positive cases, percentage of nonfatal confirmed cases with nosebleeds, and case-fatality ratio) reflects lowering of the surveillance threshold to detect cases. More patients with comparatively mild disease probably sought treatment because of the increased Lassa fever publicity and communication about available therapy; moreover, additional suspected cases were probably detected in the community through enhanced contact tracing and active case finding.

In conclusion, we cannot exclude that early 2018 represents a particularly active Lassa fever season in Nigeria, especially in Edo, Ondo, and Ebonyi. However, no available evidence indicates that higher case numbers could be attributed to new virus strains. The addition of new laboratories with growing surveillance capacities, an overall intensified response, and increasing surveillance sensitivity are likely major drivers of the high number of Lassa fever cases reported in early 2018. The weekly case numbers reported in early 2019 slightly surpass those from 2018 (https://ncdc.gov.ng/themes/common/files/sitreps/b94e459c79a59ca9d667a55539cda5db.pdf). Improved identification of Lassa fever cases in Nigeria provides the basis for epidemiologic studies of disease and effective disease control. Also, each identified case treated in isolation centers reduces the likelihood of person-to-person transmission.
